# Recruiting a National Sample of First Response Agencies to Participate in an Overdose Prevention Research Project: Randomized Controlled Trial and Feasibility Study

**DOI:** 10.2196/81743

**Published:** 2026-04-02

**Authors:** Jon Agley, Cris Henderson, Monica Nair, Dong-Chul Seo, Maria Parker, Lilian Golzarri-Arroyo, Stephanie Dickinson, David Tidd

**Affiliations:** 1 Prevention Insights, School of Public Health-Bloomington, Indiana University Bloomington, 809 E. 9th St., Bloomington, IN, 47405, United States, 1 812-855-3123; 2 Department of Applied Health Science, School of Public Health-Bloomington, Indiana University Bloomington, Bloomington, IN, United States; 3 Department of Epidemiology and Biostatistics, School of Public Health-Bloomington, Indiana University Bloomington, Bloomington, IN, United States; 4 Biostatistics Consulting Center, School of Public Health-Bloomington, Indiana University Bloomington, Bloomington, IN, United States

**Keywords:** recruitment, first responder, overdose, opioid, community engagement, PulsePoint, randomized controlled trial, RCT, naloxone

## Abstract

**Background:**

US overdose deaths continue to exceed 77,000 per year, the majority of which involve opioids. One evidence-based response to this crisis is overdose education and naloxone distribution (OEND). There is a large national (US) network of citizens and first response agencies connected through an app called *PulsePoint Respond*, who are engaged in facilitating rapid layperson cardiopulmonary resuscitation administration in cases of public emergencies. Our goal is to recruit these first response agencies to provide targeted messaging about OEND to this large subpopulation of motivated layperson responders. This study focuses on the first step: the feasibility of our national efforts to recruit first response agencies to participate in our project.

**Objective:**

This study aimed to determine whether more first response agencies were successfully recruited using materials that included preemptive correction of misperceptions about overdose and naloxone than with standard recruitment materials and to investigate the recruitment parameters observed when agencies were successfully recruited.

**Methods:**

The overall study was a randomized controlled trial in which we randomly sampled 180 first response agencies from the total set of agencies subscribing to *PulsePoint* (n=773). Agencies were randomly allocated to 3 study arms (1:1:1) with stratification for rural status. Arm 1 received standard recruitment materials, arm 2 received similar materials that directly addressed common misperceptions about overdose and naloxone, and arm 3 was recruited to serve as a control arm for later parts of the study. The primary analysis of recruitment approaches used logistic regression, contrasting arms 1 and 2. Exploratory analyses included descriptive statistics and other logistic regression models.

**Results:**

A total of 40 agencies signed memoranda of understanding to participate in the project (n=176, 22.7% of contacted agencies; n=151, 26.5% of the agencies where a point of contact had been established). We did not find evidence that the messaging contained in arm 2 significantly affected recruitment success (odds ratio 0.754, 95% CI 0.298‐1.904; *P*=.55). Likewise, arm assignment (3-way comparison) did not significantly affect the likelihood of an agency agreeing to participate. The recruitment process took a mean of 159.08 (SD 104.74) days per agency and involved 8.38 emails, 1.98 voicemails, 0.83 phone calls, and 1.23 video calls.

**Conclusions:**

Recruiting first response agencies that subscribe to *PulsePoint* for participation in a national-level OEND project appears feasible, with an anticipated participation rate between 23% and 27% of agencies solicited. Successful recruitment timelines can be lengthy and involve extensive correspondence. Since the language used in our different study arms did not have a significant effect on agency recruitment, other factors (such as individual citizen responses to messaging) could reasonably be used to select the overall language used in subsequent project recruitment materials.

## Introduction

### Background

Although US overdose death rates have decreased substantially in recent years, including a 26.9% reduction from 2023 to 2024 [1], the most recent 12-month-ending overdose death count (77,677 in January 2025) still exceeded every other overdose death count reported on or before April 2020 [2]. The majority of those overdose deaths involved opioids [1,2].

The national response to this overdose crisis is complex and multifaceted; one key component is the use of overdose reversal medications such as naloxone (eg, Narcan), which can safely reverse an opioid overdose [3]. Evidence suggests that overdose education and naloxone distribution (OEND) programs of various kinds are effective preventive mechanisms [4-9]. Questions remain, however, about the optimal ways to implement such programming on a national scale.

In our protocol for this project, we argue that an ideal opioid overdose reversal infrastructure is one in which “naloxone is present at or near the scene” of an overdose and “used as quickly as possible,” by which we imply a saturation of (1) individuals ready, willing, and able to respond to an overdose and (2) accessible overdose reversal medication [10]. This paper outlines the first step in the systematic approach by which we are working to support such saturation.

### Our Overall OEND Project

The objective of our overarching project is to partner with the “large, highly engaged system for cardiopulmonary resuscitation (CPR) that already exists nationally in the US (*PulsePoint Respond*)” to support proliferation of OEND by sending messages to *PulsePoint Respond* app users that provide information about opioid overdose, direct them to online overdose and naloxone administration training opportunities, and remind them of the importance of carrying naloxone [10]. The project involves a 2-stage process, of which the first stage is the focus of this study.

For this first phase, we randomly sampled 180 first response agencies from the total set of agencies partnered with the *PulsePoint Respond* app (n=773). We attempted to recruit those 180 agencies to participate in the second phase of our project (to send out messaging to their *PulsePoint Respond* users). The second phase, which is ongoing in conjunction with participating agencies, involves providing messaging to citizen responders subscribed to those agencies’ feeds in the *PulsePoint Respond* app. We briefly describe elements of that phase to contextualize our study, but it is not the focus of this paper.

### PulsePoint Respond

At the time of our proposal, the *PulsePoint Respond* app, developed by the nonprofit PulsePoint Foundation, had more than 1 million monthly active users receiving geotargeted alerts on their mobile devices in around 4800 communities in the United States represented by more than 700 agencies [10]. As of July 2025, the number of monthly active users had surpassed 1.37 million, and there are now more than 5500 connected communities [11].

*PulsePoint Respond* was originally launched in 2011. Now, the hundreds of agencies working with PulsePoint are located across North America [12]. “PulsePoint implementations are typically championed and led by local Fire/EMS (Emergency Medical Services) agencies” who can reach out to PulsePoint to initiate discussions about joining as a community [13]. Those agencies then use a variety of methods to encourage individual-level (eg, user) participation via community outreach.

While first response agencies can directly recruit individuals with specialized skills or professions and enroll them as “Registered CPR Responders” or “Professional Responders,” our primary interest for this project is “Public CPR Responders,” who compose the majority of users. These individuals “are typically community members trained in CPR and automated external defibrillator use and willing to assist if an incident occurs near them” [14]. Any individual with the *PulsePoint Respond* app is able to sign up as a Public CPR Responder. Public CPR responders receive “notification of nearby cardiac arrest events occurring in public places” any time they are in a PulsePoint-connected community. They can also sign up for community alerts in multiple communities and are “shown a filtered list of emergencies occurring in the community and offered notifications of public interest events such as traffic collisions and wildland fires” [14].

### PulsePoint’s Public CPR Responders

We speculate that the individuals who opt in as Public CPR Responders for the *PulsePoint Respond* app are systematically different from the general public in the sense that they have already displayed a willingness to be alerted about, and potentially respond to, emergency incidents occurring in nearby public spaces (such as an unconscious and unresponsive person). Therefore, these individuals represent promising candidates for OEND outreach. As we have noted, our overall project, of which this study is the first component, is to provide informative messaging to *PulsePoint Respond* users, alerting them to online overdose and naloxone administration training opportunities and reminding them of the importance of carrying naloxone [10]. However, the app does not have a feature by which messages can be sent to all users in the system. Instead, the highest “level” of messaging permission within the app occurs at the “agency level” (eg, subscribing to a 911 dispatch center or firehouse), which can send messages to all app users who have subscribed to that agency’s alerts.

### The Need to Recruit First Response Agencies to Accomplish Our Study

Therefore, in order to study whether our targeted OEND messaging approach results in higher percentages of community-level responders reporting that they carry naloxone or have been trained in opioid overdose response, we needed to recruit first response agencies as partners willing to send messages to their users. Unfortunately, such a recruitment process does not appear to have precedent in the literature.

There are many examples where individuals at first response agencies have been recruited to participate in research related to overdose and naloxone, particularly interviews and surveys. For example, studies have done so using email surveys to agency leaders [15,16], surveys at in-service training [17], convenience and snowball sampling for interviews [18], and email and telephone recruitment from multiple divergent sources [19]. However, we were unable to locate any systematic descriptions of studies where first response agencies were recruited to partner with researchers to conduct subsequent research tasks (as was our goal here). Given this lack of information, we developed a study of agency recruitment as part of the first phase of our overall study.

### The Value of Understanding First Response Agency Recruitment

We believe it is important to fill the gap in the literature on recruiting first response agencies as research partners for several reasons. First, clear information about agency participation rates will provide useful grounding for future studies. For example, the participation rate that one might reasonably expect from such an effort (eg, the number of agencies X required to actually achieve a participating sample Y) was unknown. While such estimates can vary by context, knowing an approximate participation rate can assist with statistical and practical study planning. Second, the overall effort required to recruit such agencies had not previously been studied or documented; obtaining a precise sense of the types of procedures and the length of time required to secure a participation agreement is critical for researchers planning a study timeline and allocating staffing time and costs.

In addition, this study provided us with an opportunity to test whether different message types were more likely to result in successful agency recruitment. As we describe in our protocol [10], “misperceptions about overdose and naloxone, as well as stigmatizing beliefs about people who use drugs, may affect both layperson and first responder willingness and interest in carrying and using naloxone,” a topic about which much has been written [19-28]. Research also suggests that inaccurate ideas about naloxone and overdose are relatively common among US adults [29]. Thus, we speculated that recruitment messages that proactively addressed concerns about OEND that were predicated on inaccurate information would be more effective than recruitment efforts that did not use this approach.

### Objectives

Our goal was for agencies to agree to participate in a structured program to send push messages (within the *PulsePoint* app) to citizen responders to provide information about opioid overdose, naloxone, and online overdose training opportunities. This study aimed to examine the feasibility of recruiting first response agencies that already subscribe to the *PulsePoint Respond* program [13], to test the efficacy of different messaging strategies, and to estimate the parameters (eg, length of time, contact quantity, and type) involved in successfully recruiting an agency. Except for one exploratory analysis, these aims were preregistered [10].

The primary hypothesis was:

- More first response agencies will be successfully recruited by study arm 2 (which included preemptive correction of inaccurate information about overdose and naloxone) than by study arm 1 (which included standard recruitment messaging).

Exploratory analyses were also conducted to answer the following questions:

- Were there significant differences in recruitment success between any of the study arms (including arm 3, which was a control arm)? This exploratory analysis was not preregistered.
- What was the mean length of time between establishing an initial point of contact (POC) and successfully establishing a memorandum of understanding (MOU) to participate in the project?
- What were the mean numbers and types of correspondence associated with successfully establishing an MOU to participate in the project?

In our protocol, we also explicitly noted that “regardless of the results of null hypothesis significance testing, [we were also interested in obtaining] an overall sense of recruitment feasibility” [10].

## Methods

### Design, Setting, and Eligibility

This study was a randomized controlled trial using a parallel-group design and a 1:1:1 allocation ratio with postsampling allocation by rurality. Eligible participants were all first response agencies in the United States that subscribed to the *PulsePoint Respond* app as of January 30, 2024 (n=773). At the sampling stage, agencies were considered ineligible and replaced if they had previously worked with our study team on a similar project.

### Sampling, Allocation, and Blinding

A sample of 180 agencies was drawn based on the primary hypothesis contrasting 2 of the 3 study arms. With 60 agencies per arm, 80% power, and a 2-tailed α of .05, we had the ability to detect a proportional difference of at least 0.25 (ie, a 15-agency difference between arms) [10].

A study statistician not involved with the recruitment process (LGA) produced a random computer-generated sequence of numbers and sent it to JA, who then overlaid it on the population of eligible agencies, which had been provided to our study team by the *PulsePoint* Foundation in no particular order. As prespecified, the 180 agencies with the lowest random numbers were considered to be the initial sample.

Of those agencies, 2 were excluded for active or past overdose training work with the study team on different projects, 2 were invalid entries (eg, test cases that had been left in the dataset), and one was located outside the United States. The 5 agencies with the next lowest numbers in the original sequence were then included in the sample.

Then, the rural status of each agency was determined using the procedure described in *Supplemental File: Rurality Determination Method* [30]. As planned, allocation to study arms was accomplished by allocating the lowest third of rural agencies and the lowest third of nonrural agencies to arm 1, then the middle thirds to arm 2, and the remainder to arm 3. Because allocation was directly determined by the initial random number sequence, one can infer that allocation was concealed until assignment.

Agencies were blinded to arm assignment; they were informed about the overall project and the total number of agencies but not that some agencies received different materials or were asked to complete slightly different tasks.

### Intervention and Comparator Conditions

#### General Recruitment Procedures and Content Design

Outreach to all agencies as part of the recruitment process involved combinations of phone calls, emails, and voicemails. The initial points of contact were obtained through a search for the agencies’ publicly available data. The initial goal was to establish a POC (eg, a person at the agency familiar with *PulsePoint* and able to discuss the project with our team) [10]. Once the POC had been identified for an agency, we requested a videoconference (~10 min) with them to discuss the project using an arm-specific slide deck. We also provided arm-specific flyer sets in PDF format (2 variants targeted at the agencies and 2 variants that the agencies could prospectively use for outreach to citizens in their coverage areas). Except for ad hoc communication about specific topics, our team used a suite of template emails and voicemails depending on the state of recruitment (see *Supplemental File: Email and Voicemail Templates* [30]).

All materials (including push message examples) provided to agencies were codeveloped with marketing professionals at IU University Communications and Marketing Creative & Web Team (CWT). Our core project team developed initial materials that were then iteratively refined by personnel at the CWT. The CWT personnel were asked to document the key gray and academic literature used to inform their decision processes around push/SMS text messaging best practices [31-33], community-based overdose and naloxone-related communication [34-37], general marketing language [38], visuals to address harm reduction [39,40], and strategies to write about harm reduction [41-45].

#### Study Arm 1

**Figure 1.**
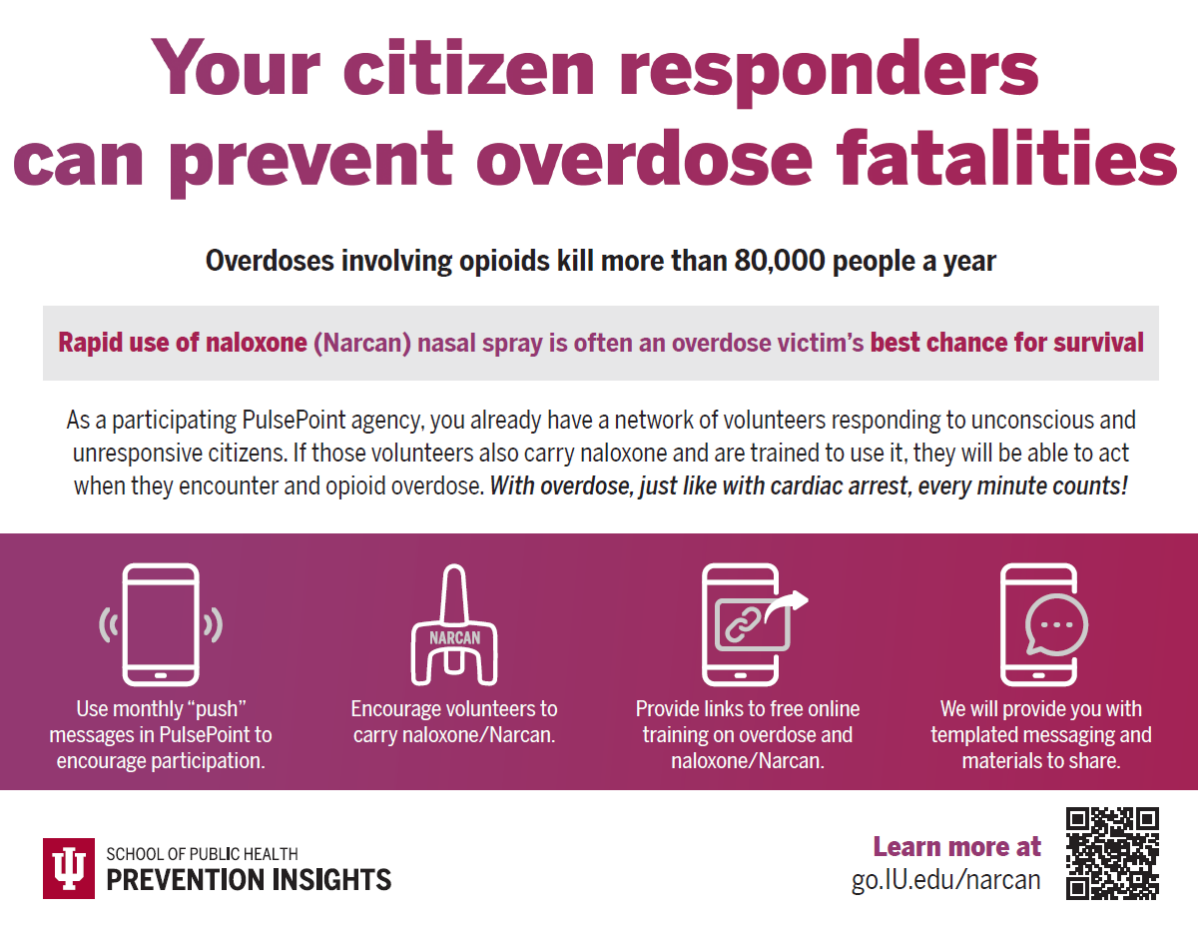
Sample agency flyer for study arm 1.

Arm 1 was designated the “standard” recruitment arm, which focused on general messaging to encourage engagement with our program, such as (Figure 1):

- “Your citizen responders can prevent overdose fatalities.”
- “As a participating *PulsePoint* agency, you already have a network of volunteers responding to unconscious and unresponsive citizens. If those volunteers also carry naloxone and are trained to use it, they will be able to act when they encounter an opioid overdose. With an overdose, just like with cardiac arrest, every minute counts!”
- “The US Surgeon General recommends: ‘Be Prepared. Get Naloxone. Save a Life.”

Agencies were asked to commit to sending a “push” message through the *PulsePoint Respond* app once per month for 1 year. They were also asked to send 3 sets of 2 data collection messages (baseline, 6 months, and 12 months) asking participants to indicate whether they had been trained and carry naloxone. When agencies verbally committed to the project, they were provided with an MOU outlining these procedures. Upon signing the MOU, agencies were provided with instructions and push message templates (to be shared once the citizen portion of the study is completed in 2026). All recruitment materials for arm 1 can be found in the *Supplemental File: Arm 1 Recruitment Materials* [30].

#### Study Arm 2

**Figure 2.**
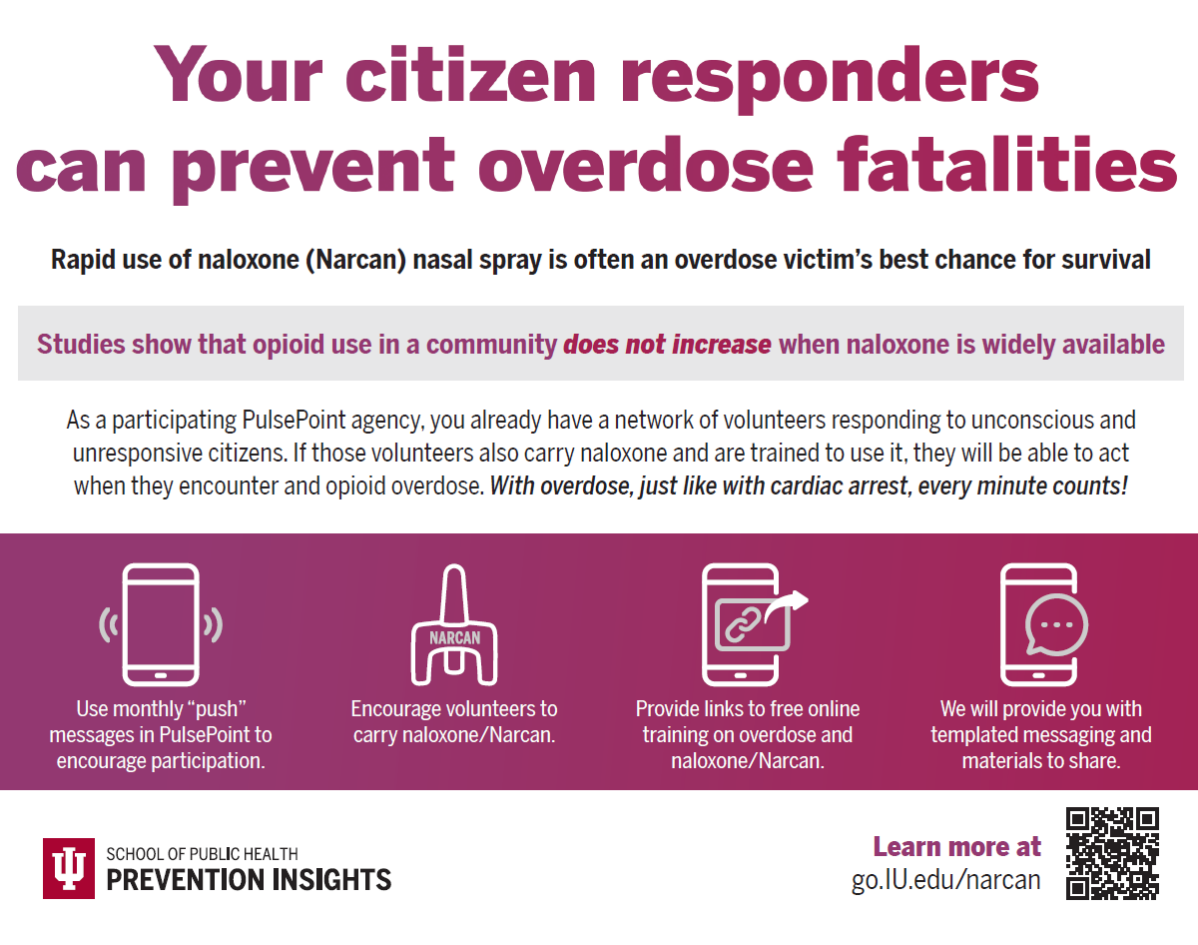
Sample agency flyer for study arm 2.

Arm 2 was designed to use most of the recruitment principles from arm 1 while preemptively providing brief statements reflecting the current state of scientific knowledge to counteract common misconceptions about opioid overdose and naloxone [29]. Examples of such statements include (Figure 2):

- “Studies show that opioid use in a community does not increase when naloxone is widely available” [46-50].
- “Studies show that most people who are revived after an overdose do not overdose again” [51-54].

Except for messaging changes described above, other recruitment procedures were similar to arm 1 (see *Supplemental File: Arm 2 Recruitment Materials* [30]).

#### Study Arm 3

Arm 3 was designed as a control arm for subsequent studies within this project. It used the standard messaging from arm 1 except that agencies were only recruited to send the sets of data collection messages. The modified slide deck for this arm is available in *Supplemental File: Arm 3 Recruitment Slide Deck* [30].

### Outcomes and Covariates

The following variables were used in our analysis:

- Agency recruitment: The “ratio of recruited agencies (MOU) to total agencies with which active communication has been established,” [10] (meaning that a POC was reached). This was the primary preregistered outcome.
- Raw MOU completion: The ratio of agencies with a completed MOU to the total denominator within a study arm. This was a secondary exploratory outcome.
- Recruitment timeline: The “length of time between initial contact and agreement to participate (MOU),” [10] measured in days. This was a secondary preregistered outcome.
- Dose-response metric: The “mean number and types of correspondence associated with agreement to participate and completion of an MOU” [10]. This was a secondary preregistered outcome.

### Changes to Trial Protocol After Preregistration

We made 3 specific decisions about how to analyze and report this trial following preregistration, which we detail subsequently. We also documented all other events that might plausibly have affected the results of the study but that we considered to be minor or ecological issues. That documentation was timestamped at the time it was produced and is available in the *Supplemental File: Other Considerations for Study Interpretation* [30].

First, we originally planned to include agency rurality (and vote share derived from ZIP codes) as covariates in our primary outcome analysis. As we learned throughout the data collection process, many of the agencies in the sample covered broad mixtures of rural and nonrural spaces, sometimes with agencies overlapping in the same ZIP code for different service types. As described in *Supplemental File: Rurality Determination Method* [30], we were able to calculate a rough estimate of rural status to accomplish stratified allocation. However, after learning more from agencies about their operational procedures and collaborative norms, it was not clear to us that including these geography-based covariates would accomplish the goal of reducing error variance [55]. Particularly since agencies had already undergone stratified allocation to study arms by rural status, we chose to exclude these covariates from further analysis.

Second, we originally proposed to treat “agreement to participate” (informal) and “signing an MOU to participate” as having the same meaning in our primary outcome variable (agency recruitment). After observing that a high number of agencies expressed interest in participating but ultimately transitioned to refusals (eg, appeared to indicate that they would participate, but ultimately did not sign an MOU), we determined that such a designation would be misleading. To more conservatively estimate recruitment feasibility, we chose to adopt the more stringent requirement (a signed MOU) as the recruitment success metric.

Third, we originally stated that we would conduct separate analyses at 6 and 12 months postcorrespondence. After conducting the project, it became clear that 6-month analyses would not yield useful information, particularly since recruitment of specific groups of agencies was temporarily paused for various ecological reasons within the 12-month period (eg, fires, hurricanes, and floods). Further, agencies were only ever informed of a final deadline (12 mo).

### Statistical Analysis

Analyses of agency recruitment used unadjusted logistic regression, both on the full sample and, separately, on the subsample of agencies for which correspondence had been achieved (adjustment was not necessary due to randomization). There were no missing data to address for those analyses. Those analyses, as well as measures of central tendency and distribution for all variables, were calculated using SPSS (version 29; IBM Corp).

### Ethical Considerations

The agency recruitment processes within our study were reviewed by the Indiana University Institutional Review Board and determined not to meet the federal definition of research involving human subjects (review board record number 20218). Results are reported in a manner consistent with the CONSORT (Consolidated Standards of Reporting Trials) 2025 guidelines [56] (Checklist 1).

## Results

### Participants

An initial sample of 180 agencies was randomly drawn from the list of 773 agency subscribers. As described in the Methods section, 5 of those agencies were removed from the sample due to ineligibility criteria and then replaced. Each agency’s rural status was determined to the best degree possible, and then agencies were allocated with stratification for rural status to study arms (60:60:60). During the rolling 12-month recruitment period (May 2024 to May 2025), agencies were replaced and resampled if they:

- Confirmed that they were no longer a *PulsePoint* subscriber (n=22)
- Were completely inactive in the *PulsePoint Respond* app and did not respond to any inquiries, signifying likely nonsubscription (n=5)
- Were part of another agency that was also listed in *PulsePoint* (eg, a single agency had 2 different “listings” in the app, but they were not actually separate) (n=9)

**Figure 3.**
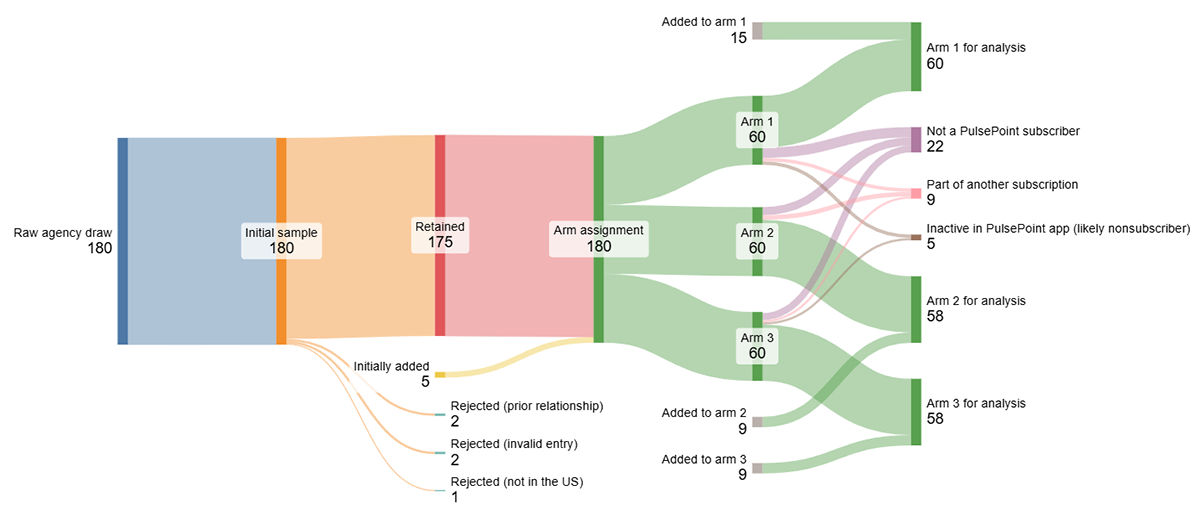
CONSORT (Consolidated Standards of Reporting Trials) (Sankey) study diagram.

Each of these instances was documented in real time (see *Supplemental File: Sampling Documentation [Redacted Names]* [30]). In one instance, a case of “merged” agencies was identified very near to the end of recruitment, and so those final cases were not resampled, meaning that the final denominators by arm were 60:58:58 (Figure 3).

### Recruitment

We were able to establish POCs for the majority of agencies in study arm 1 (49/60, 81.7%), study arm 2 (56/58, 96.6%), and study arm 3 (46/58, 79.3%) (see Table 1). In most cases, failure to establish a POC reflected the inability to connect with any individual at the agency after the prescribed number of attempts; however, in some cases, the agency was also unable to determine who the correct POC would be.

**Table 1.** Recruitment statistics.

Study arm	Point of contact established, n	MOU^a^ signed, n
1 (n=60)	49	12
2 (n=58)	56	11
3 (n=58)	46	17

a MOU: memorandum of understanding.

A total of 40 agencies signed MOUs to participate in the project (22.7% [176] of total agencies; 26.5% [151] of the agencies where a POC had been established). We did not find evidence that the nature of the messaging contained in study arms 1 or 2 significantly affected recruitment success (odds ratio 0.754, 95% CI 0.298‐1.904; *P*=.55). We conducted exploratory analyses to examine 3-way comparisons (including the control arm) between agencies with established POCs and, separately, between all agencies, and likewise found that arm assignment did not significantly affect the likelihood of an agency signing an MOU to participate (see Table 2).

**Table 2.** Results of primary and exploratory analyses.

Comparison	OR^a^	95% CI	*P* value
Point of contact established (2-way comparison)^b^			
Arm 2 vs arm 1	0.754	0.298‐1.904	.55
Point of contact established (3-way comparison)			
Arm 2 vs arm 1	0.754	0.298‐1.904	.55
Arm 3 vs arm 1	1.807	0.746‐4.377	.19
Arm 3 vs arm 2	2.398	0.984‐5.843	.054
All agencies in sample (3-way comparison)			
Arm 2 vs arm 1	0.936	0.376‐2.330	.89
Arm 3 vs arm 1	1.659	0.710‐3.874	.24
Arm 3 vs arm 2	1.772	0.745‐4.213	.20

a OR: odds ratio.

b Primary analysis for this study.

The length of the recruitment period, defined as the number of days between the initial contact attempt and our receipt of the signed MOU, was 159.08 (SD 104.74; range: 18-365) days (see Table 3).

**Table 3.** Length of recruitment period.

Study arm	Number of days, mean (SD)	Range of days
1 (n=12)	164.75 (113.47)	18‐341
2 (n=11)	209.27 (130.82)	27‐365
3 (n=17)	122.59 (63.42)	38‐249
Combined (n=40)	159.08 (104.74)	18‐365

Between the initial contact attempt (to establish a POC) and the time when a signed MOU was received for each agency, our study team sent an average of 8.38 emails, left 1.98 voicemails, and conducted 0.83 phone calls, in addition to holding 1.23 video calls during which we shared the recruitment slide deck for the project. These figures represent outgoing messages only and do not include messages received by the team.

Three agencies in the final sample were single agencies resulting from “merges” of multiple agencies that were listed separately in the *PulsePoint* app (see Figure 3). Therefore, Table 4 also provides descriptive statistics that exclude those agencies (sensitivity analysis), because some of the correspondence that reasonably might be attributed to recruiting these agencies may have been tagged as being part of other agency IDs, so we share these additional numbers for purposes of transparency.

**Table 4.** Instances of outgoing recruitment correspondence.

	Number of emails, mean (SD)	Number of voicemails, mean (SD)	Number of phone calls, mean (SD)	Number of video calls^a^, mean (SD)
Study arm				
1 (n=12)	8.17 (4.09)	2.42 (1.73)	1.17 (1.11)	1.50 (0.67)
2 (n=11)	10.09 (3.94)	2.55 (1.57)	0.91 (1.04)	1.18 (0.40)
3 (n=17)	7.41 (3.08)	1.29 (1.40)	0.53 (0.62)	1.06 (0.24)
Combined (n=40)	8.38 (3.72)	1.98 (1.62)	0.83 (0.93)	1.23 (0.48)
Study arm (sensitivity)				
1 (n=12)	8.17 (4.09)	2.42 (1.73)	1.17 (1.11)	1.50 (0.67)
2 (n=9)^b^	10.22 (4.32)	2.78 (1.48)	1.00 (1.12)	1.22 (0.44)
3 (n=16)^b^	7.69 (2.96)	1.38 (1.41)	0.56 (0.63)	1.06 (0.25)
Combined (n=37)^b^	8.46 (3.74)	2.05 (1.61)	0.86 (0.95)	1.24 (0.49)

a Each agency was required to have at least one video call to discuss study materials, so the lowest possible mean value was 1.

b Data from agencies that formed as part of “merges” were excluded

## Discussion

### Summary of Results

This study was designed to examine the feasibility of recruiting first response agencies that already subscribe to the *PulsePoint Respond* program to participate in a project focused on increasing OEND on a national scale in the United States. The randomized controlled trial design also allowed us to test whether proactively addressing common misperceptions about overdose and naloxone increased the number of agencies successfully recruited compared with standard recruitment messaging.

We did not find any evidence that the type of messaging included in recruitment materials (standard vs addressing misperceptions) had an effect on whether an agency was successfully recruited to participate in our project. In exploratory analyses, we likewise did not observe any pairwise differences between any of the study arms in terms of recruitment success. However, we found that recruiting agencies to participate in an OEND project was feasible, successfully recruiting 40 of the 176 agencies that we attempted to recruit from the national *PulsePoint* subscriber database.

### Other Useful Findings

Even though our team understood from the outset that we would need to establish POCs for each agency, we were surprised by the average amount of time it took to recruit agencies for our project. While there were outlier agencies in each study arm that were recruited quickly (see range data in Table 3), the average recruitment process lasted a little longer than 5 months per agency. Based on our records, the factors contributing to this range of time seemed to vary. In some cases, it took a long time to identify the person at an agency who worked with *PulsePoint Respond*, but recruitment proceeded quickly thereafter. In other cases, we identified a POC quickly, but other individuals within the agency (eg, supervisors) or outside the agency (eg, legal counsel) needed to review issues around OEND, the nature of the project, and data privacy at greater length. In yet other cases, the recruitment process generally proceeded as planned, but each step took extra time due to first responders’ need to prioritize ongoing emergencies such as wildfires.

Thus, depending on staffing size and capacity, we note the importance of allocating commensurate levels of time and effort at the front end of large-scale agency recruitment efforts to allow for this process to unfold. At the same time, in many cases, higher numbers of contact attempts reflected greater difficulty in determining which person in a given agency worked directly with the *PulsePoint* interface.

We also expressed concern at the outset that agencies randomized to the control arm might not participate in the project because that arm had fewer direct benefits to the community (eg, sending out push messages linking citizens to resources and training) [10]. However, agencies were just as likely to be recruited for study arm 3, with many of them expressing interest in the data that would be obtained through the control arm data collection regarding the current levels of overdose training and education, and the percentages of citizens regularly carrying naloxone.

### Limitations and Alternative Interpretations

Multiple agencies were removed and replaced from the sample over the course of the project. In some cases, this resulted from agency “merges,” where more than one agency was listed separately in the sampling frame, and we only learned after establishing communication that the agencies had the same controlling agreement related to *PulsePoint*. In other cases, participating agencies in different study arms were in close proximity to each other. In both types of situations, this may have resulted in some cross-exposure between study arms, but the numbers are insufficient to have affected the analytic results.

There were also multiple ecological events, particularly major natural disasters, that occurred throughout the study period. These were equally likely to have affected any given study arm but may have reduced the overall recruitment rate for the study as a whole. It is also possible that the overall recruitment rate was affected by other overdose prevention efforts, such as naloxone leave-behind programs [57]. Like ecological events, such effects would have been randomly distributed between study arms, but recent informal discussions between a first response coalition from a separate project and one study author (DCS; see more about his other project here [58,59]) found the potential for multiple overdose response programs to be perceived as competing priorities.

Still, this study is likely to have a high level of generalizability to first response agencies that subscribe to *PulsePoint*, but whether those agencies, in turn, are systematically different from other first response agencies in the United States or elsewhere is unclear. Caution should therefore be used in inferring these findings outside of the study frame.

The lack of a significant finding for the recruitment messaging is limited to effects on agency recruitment. In subsequent studies as part of this overall project, we will determine whether there is an effect—or not—on individual layperson responders depending on the type of messaging available through their study arm, but this component of the study does not address those differences.

Finally, this study was not designed to compare recruitment in arm 3 against the active study arms (the control arm was designed to facilitate individual-level analyses in subsequent parts of the project). Exploratory analyses suggested that none of the study arms was significantly different in terms of agency recruitment. However, the raw count for the control arm was descriptively higher than for the other arms. Based on statistics alone, we cannot infer that this difference is meaningful, but we note that our recruitment discussions often touched on the low levels of staffing and high workload in first response agencies. Thus, we think it is plausible, though not established by this study, that there may be a small recruitment advantage for the control arm (on the basis of a reduced “ask” for participation) that we were underpowered to detect.

### Conclusions

This study demonstrated that it is feasible to recruit first response agencies that subscribe to *PulsePoint* to participate in an OEND project, with an anticipated recruitment success rate between 23% and 27%. The recruitment process was relatively involved, lasting slightly more than 5 months per agency on average and requiring multiple correspondence efforts across several communication platforms. Incorporating messaging designed to address inaccurate information about overdose and naloxone into messaging materials did not affect recruitment success. Supplemental qualitative analyses of recruitment materials and interviews with agency personnel about the recruitment process are underway and will provide additional clarifying information about how to optimize recruitment.

## Supplementary material

10.2196/81743 Checklist 1 CONSORT 2025 checklist.

## Abbreviations

CONSORT: Consolidated Standards of Reporting Trials
CPR: cardiopulmonary resuscitation
MOU: memorandum of understanding
OEND: overdose education and naloxone distribution
POC: point of contact
